# The lifespan extension effects of resveratrol are conserved in the honey bee and may be driven by a mechanism related to caloric restriction

**DOI:** 10.18632/aging.100474

**Published:** 2012-07-31

**Authors:** Brenda Rascón, Basil P. Hubbard, David A. Sinclair, Gro V. Amdam

**Affiliations:** ^1^ Department of Chemistry, Biotechnology and Food Science (IKBM), Norwegian University of Life Sciences (UMB), Ås N-1432, Norway; ^2^ Departments of Pathology and Genetics, Harvard Medical School, Boston, MA, 02115, USA; ^3^ School of Life Science, Arizona State University, Tempe, AZ 85287-4501, USA

**Keywords:** hyperoxia, learning performance, aging, lifespan, Apis mellifera, resveratrol

## Abstract

Our interest in healthy aging and in evolutionarily conserved mechanisms of lifespan extension prompted us to investigate whether features of age-related decline in the honey bee could be attenuated with resveratrol. Resveratrol is regarded as a caloric restriction mimetic known to extend lifespan in some but not all model species. The current, prevailing view is that resveratrol works largely by activating signaling pathways. It has also been suggested that resveratrol may act as an antioxidant and confer protection against nervous system impairment and oxidative stress. To test whether honey bee lifespan, learning performance, and food perception could be altered by resveratrol, we supplemented the diets of honey bees and measured lifespan, olfactory learning, and gustatory responsiveness to sucrose. Furthermore, to test the effects of resveratrol under metabolic challenge, we used hyperoxic environments to generate oxidative stress. Under normal oxygen conditions, two resveratrol treatments—30 and 130 μM—lengthened average lifespan in wild-type honey bees by 38% and 33%, respectively. Both resveratrol treatments also lengthened maximum and median lifespan. In contrast, hyperoxic stress abolished the resveratrol life-extension response. Furthermore, resveratrol did not affect learning performance, but did alter gustation. Honey bees that were not fed resveratrol exhibited greater responsiveness to sugar, while those supplemented with resveratrol were less responsive to sugar. We also discovered that individuals fed a high dose of resveratrol—compared to controls—ingested fewer quantities of food under *ad libitum* feeding conditions.

## INTRODUCTION

The decline in brain function that accompanies senescence in diverse organisms and the mechanisms that underlie this dysfunction are of great interest. Aging intervention strategies, such as genetic manipulation and dietary restriction, have shown that lifespan extension is possible e.g. [1]. However, enhanced longevity coupled to brain deterioration is an undesirable combination that warrants further research into the prolongation of health span. Our previous experiments in the honey bee have revealed that hyperoxic environments can mimic normal patterns of aging dysfunction, which include increased mortality and learning performance decline (Rascon et al., submitted). In the current study, we tested whether we could promote healthy aging in the honey bee via the administration of resveratrol.

Resveratrol is a plant polyphenol with reported lifespan extension effects in some [2-4], but not all studies [5, 6]. This particular polyphenol is a well-known activator of Silent Information Regulator 1 (SIRT1), which is thought to mediate the beneficial effects of caloric restriction [7, 8]. Resveratrol also elicits neuroprotective effects in vertebrate cell lines [9-12] and prevents the decline of locomotory function in fish [3]. In the brains of healthy rats, resveratrol increases the activity of antioxidants such as superoxide dismutase and catalase, and decreases the level of oxidative stress [13]. The purported, beneficial effects of resveratrol also extend to cognitive performance. For instance, resveratrol can reverse cognitive deficits and maintain memory in aged rats [14]. Also, rats suffering from traumatic brain injury [15] or demonstrating Parkinsonian characteristics [16], benefit from resveratrol treatment. However, it is unclear whether resveratrol can improve learning performance in healthy animals.

With some exceptions, most of the studies that demonstrate a resveratrol-dependent lifespan extension rely on solitary model species such as yeast, worms, and fruit flies [2-4]. To date, only one study of social animals— mice fed either a high fat diet or fed every other day—has showed improved survival in response to resveratrol [17]. In the present study, we investigated the effects of resveratrol on lifespan and learning performance in a social animal with a well-established neurobiological pedigree: the honey bee.

Briefly, the characteristic signatures of honey bee social life are the caste differences between the functionally sterile worker and highly fecund queen, and the ontogenetic specialization of tasks undertaken by worker bees. These complexities often prevent the application of many well-known evolutionary theories of aging to the honey bee [18]. For instance, explanatory paradigms such as life history theory, which postulates that the pressure of natural selection on survival falls after reproductive capacity has been reached, cannot adequately explain the aging of sterile worker honey bees. Furthermore, the disposable soma theory, which states that natural selection will not favor investment into the soma if extrinsic mortality rates are high [19], is applicable to worker honey bees only for part of their lives. For example, during nursing tasks, workers experience low hazard and low mortality, but the switch to foraging tasks outside of the nest increases hazard and mortality. However, if honey bee society is viewed as a superorganism, the worker honey bee can be considered the disposable social caste due to their high numbers, short lives, and inability to reproduce. From this standpoint, there should be evolutionary constraints on worker lifespan. But is this so? Is worker lifespan fixed or can its limits be extended?

At present, there is no published work on the effects of resveratrol in the honey bee. To achieve insight, we combine resveratrol administration with the application of metabolic stress and the classic neurobiological paradigm of olfactory conditioning to examine whether resveratrol can: 1) lengthen lifespan in the honey bee, 2) affect brain function, and 3) affect food perception (gustation) and consumption. In this study, we demonstrate that resveratrol elicits life extension in wild-type honey bees, alters gustation, and food consumption.

## RESULTS

### Learning and gustatory performance tests

All sensory and gustatory tests were evaluated in normoxic conditions after honey bees had spent five days in the laboratory under resveratrol treatment(refer to materials and methods).

#### Learning performance

**Figure 1:**
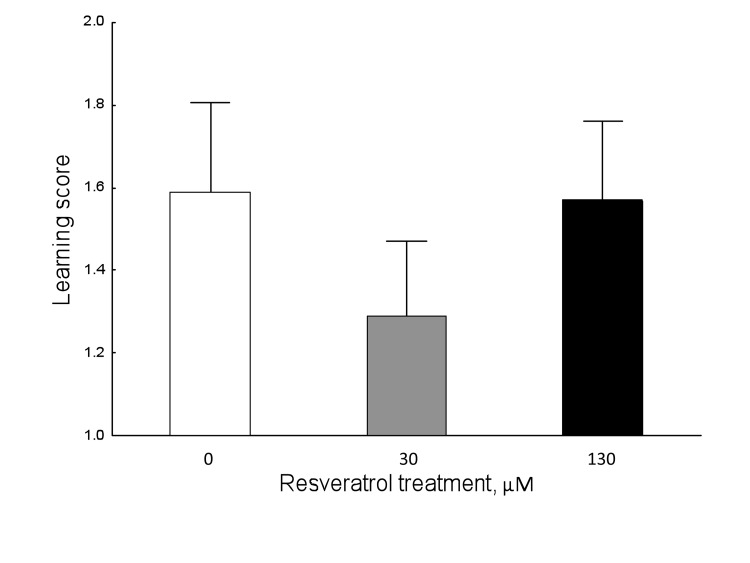
Resveratrol does not affect learning performance in 9-day-old honey bees in normoxic conditions (Kruskal-Wallis ANOVA: H ( 2, N= 350) =1.529602 P =0.465). Data shown as mean±SE.

Learning performance was not affected by resveratrol supplementation (Figure 1; Kruskal-Wallis ANOVA: H (2, N= 350) =1.529602 P=0.465).

#### Gustatory responsiveness

**Figure 2:**
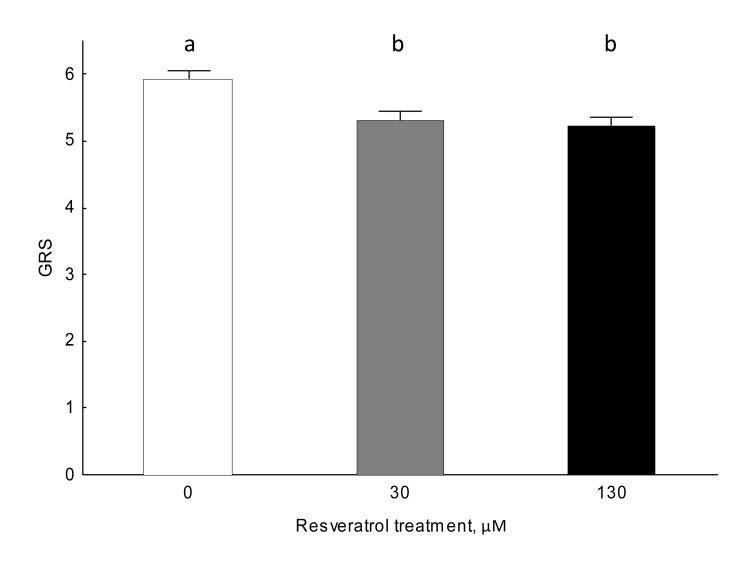
Gustatory responsiveness is altered by resveratrol in 9-day-old honey bees in normoxic conditions (Kruskall-Wallis ANOVA: H (2, 566)=11.363, N=578, P=0.003). Data shown as mean±SE. Identical letters indicate that groups are not significantly different from one another at an alpha significance level of 5% for the Mann-Whitney U test.

Resveratrol significantly influenced gustatory responsiveness (Figure 2; Kruskall-Wallis: H (2, 566)=11.363, N=578, P=0.003). The gustatory responsiveness of 30 and 130 μM resveratrol-fed individuals significantly differed from controls (Mann-Whitney U test; 0 vs 30 μM: [U=14117, N=168, N=193, P=0.017] 0 vs 130: [U=14061, N=168, N=205, P<0.001]). Resveratrol-supplemented honey bees showed lower gustatory responsiveness scores, while unsupplemented control animals showed higher scores (Figure 2). There were no significant differences in gustatory responsiveness between 30 and 130 μM resveratrol-fed honey bees (Mann-Whitney U test: U=18847.50, N=193, N=205, P=0.38).

### Effect of resveratrol on honey bee lifespan and food consumption

**Figure 3:**
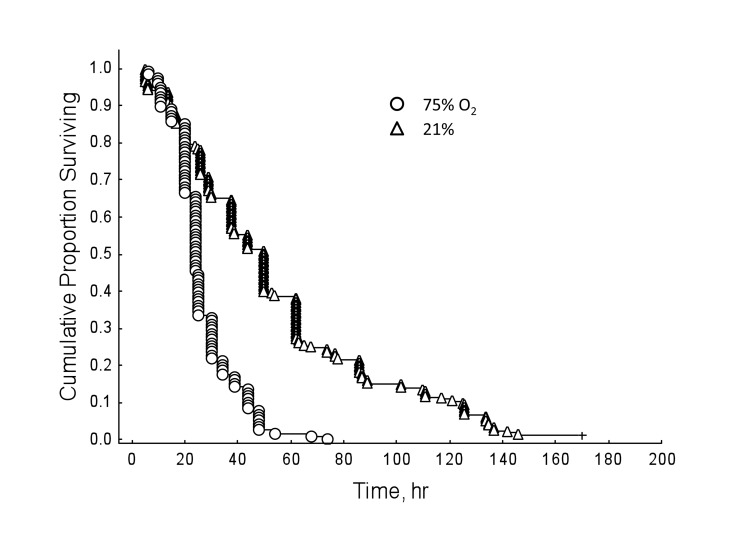
Honey bees show decreased survival rates in hyperoxia regardless of resveratrol dosage, compared to normoxic controls (Cox's F-test: F(328, 238)=2.69, P<0.01).

**Figure 4:**
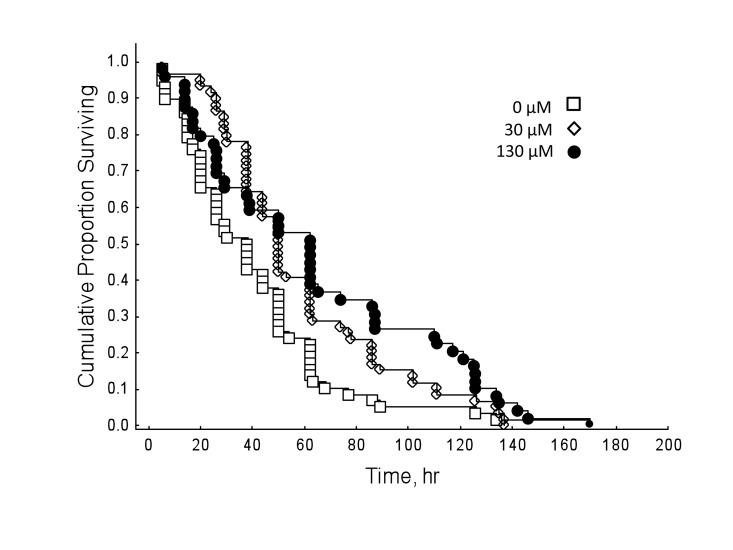
Under normal oxygen conditions, resveratrol extends lifespan in 9-day-old honey bees (Multi-group survival test: χ2 =11.72, df=2, N=130, P<0.01). Both 30 and 130 μM resveratrol treatments increased average lifespan in wild-type honey bees by 38% and 33%, respectively. Resveratrol treatments also lengthened maximum and median lifespan. Differences considered significant if P < 0.05.

**Figure 5:**
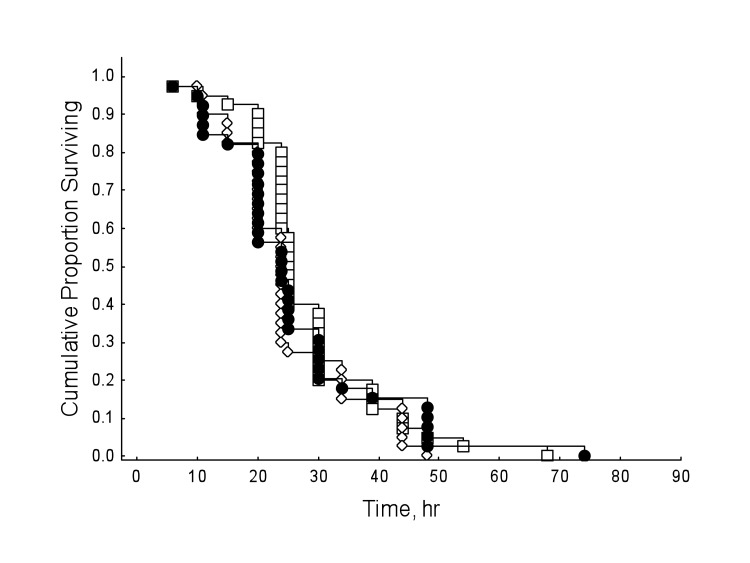
The life-lengthening effect of resveratrol, previously seen in normoxia, is abolished under sub-optimal conditions of hyperoxia (Multi-group survival test: χ2 =4.08, df=2, N=60, P=0.130).

Honey bees reared in hyperoxia compared to normoxic controls showed decreased survival rates regardless of drug dosage (Figure 3; Cox's F-test: F(328, 238)=2.69, P<0.01). However, under normal oxygen conditions, resveratrol significantly lengthened lifespan in honey bees (Figure 4; Multi-group survival test: χ2 =11.72, df=2, N=130, P=0.003). The two resveratrol treatments of 30 and 130 μM increased average lifespan by 38% and 33%, respectively (Figure 4). Both resveratrol treatments also lengthened maximum and median lifespan. Hyperoxia abolished the life extension effect of resveratrol previously seen in normoxic controls (Figure 5; Multi-group survival test: χ2 =4.08, df=2, N=60, P=0.130).

#### Food consumption

**Figure 6:**
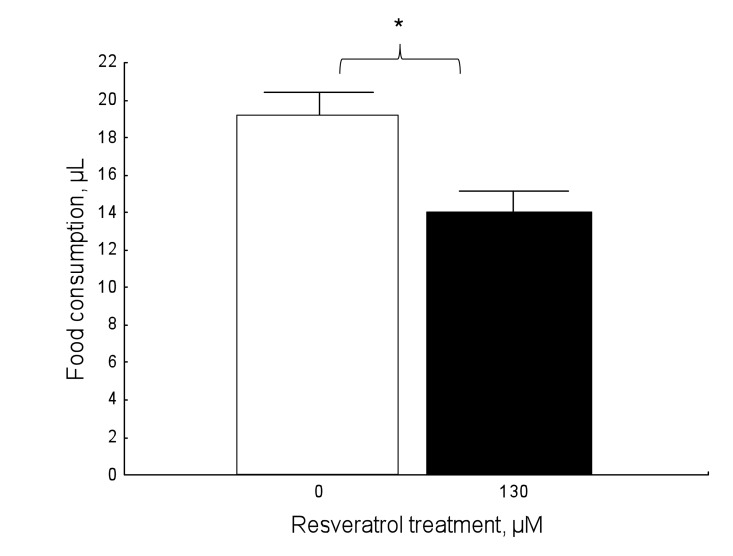
Resveratrol affects food consumption in a dose-dependent fashion in 9-day-old honey bees in normoxic conditions (Mann-Whitney: U=775, N1=49, N2=46, P=0.008). Data shown as mean±SE. The star denotes significant differences between the groups.

Resveratrol affected food consumption compared to controls (Figure 6; Mann-Whitney: U=775, N1=49, N2=46, P=0.008). Resveratrol-supplemented honey bees significantly reduced their food consumption, compared to unsupplemented individuals.

## CONCLUSIONS AND DISCUSSION

### Resveratrol and lifespan

Prior studies on yeast, worms, and fruit flies [2, 4] (Figure 2) have demonstrated a life-prolonging effect of resveratrol. However, the resveratrol-dependent lifespan effects in yeast, flies, and worms have been called into question by the experimental results of some studies [5, 6]. Nevertheless, several other inquires have provided support for the original findings of Howitz et al and Wood et al [20-23]. Our results in the honey bee reveal that resveratrol enhances lifespan under normal oxygen conditions and are thus consistent with the original reports of Howitz et al. and Wood et al.

Resveratrol is purported to function as a signaling molecule [24]. Various studies also show resveratrol may act as an antioxidant [13, 25, 26] or a pro-oxidant DNA mutagen [25, 27, 28]. In the present study, hyperoxia abolished the lifespan extension effect of resveratrol seen under normoxic conditions and failed to act as a compensatory agent in restoring lifespan to previous lengths seen in ambient oxygen (Figure 3). That said, the tension of hyperoxia (75% O2) we used was near pure oxygen, which is known to induce an array of deleterious physiological responses in *Drosophila*, including increased levels of protein carbonyl formation [29], nervous system destruction [30, 31], and neurodegeneration [32]. Furthermore, resveratrol is susceptible to *in vitro* oxidation under normal oxygen conditions [33], so it is possible that the hyperoxygenated environment used in these experiments altered the functionality of resveratrol enough to prevent lifespan extension. Lastly, the possibility that stress may account for the lack of a resveratrol-dependent lifespan extension in other studies is worth mentioning. However, more replicates in animal models would be necessary to confirm this speculation.

### Neurophysiological effects of resveratrol

Several studies in neurons, cells, and a short-lived fish species have demonstrated that resveratrol and some of its derivatives can elicit neuroprotective effects [3, 9-12] (Figure 4). In the fish, *Nothobranchius furzeri*, resveratrol delayed the onset of locomotory and learning performance decline during aging [3]. Furthermore, resveratrol increased the activity of antioxidants and decreased oxidative stress in rat brains [13]. Resveratrol can also preserve brain function in neurologically impaired rats [15, 16]. But it is unknown whether resveratrol can improve learning performance in healthy animals. As a result, our aim was to examine this question in wild-type worker honey bees. We hypothesized that resveratrol would enhance learning performance in the honey bee.

In contrast to our hypothesis, resveratrol did not improve the learning performance of honey bees in a significant way. However, resveratrol treatment did significantly change the gustatory responsiveness score (Figure 5). Unsupplemented honey bees exhibited greater responsiveness to sugar during this test, while animals supplemented with resveratrol were less responsive to sugar. This finding is particularly interesting because observations in natural settings reveal that gustatory responsiveness remains intact throughout aging in the honey bee, a response we previously replicated in the laboratory using hyperoxia (Rascón et al., in progress).

We hypothesized that an altered gustatory response score could indicate that resveratrol was eliciting a satiety effect on honey bees. This prompted us to measure individual food consumption in resveratrol-supplemented and unsupplemented subjects.

### Effects of resveratrol on food consumption

Resveratrol is thought to slow aging by mechanisms that may be related to caloric restriction. Caloric restriction is widely known to increase lifespan across organisms, as well as prevent the onset of diseases associated with old age [1]. Several studies in diverse organisms indicate that sirtuins may facilitate the effects of caloric restriction [34-36]. Added to this, the lifespan extension effect of resveratrol appears to depend on the activation of sirtuins [37], which play known roles in energy metabolism [38-40]. Most [6, 35, 41, 42], but not all studies [43], demonstrate that the overexpression of Sir2 homologs extends lifespan. Notably, the overexpression of SIRT1 in mice produces phenotypes reminiscent of caloric restriction [44]. A recent bioinformatics study that compared the gene expression profiles of species subjected to caloric restriction, Sir2 overexpression, and resveratrol administration discovered that 23 genes involved in stress, metabolism, and growth were conserved in response to caloric restriction and resveratrol [45]. This suggests that resveratrol supplementation and caloric restriction share some common molecular responses.

In the present study, resveratrol-supplemented individuals showed a significant reduction in food consumption under *ad libitum* feeding conditions, compared to unsupplemented controls, which consumed more food (Figure 6). To the best of our knowledge, this has never been demonstrated for the honey bee, but our results are consistent with a study on mouse lemurs in which resveratrol decreased food intake by increasing satiety [46]. Furthermore, mice with an additional copy of SIRT1 exhibit decreased food intake [47] and hypothalamic SIRT1 seems to regulate food consumption in rats [48]. Resveratrol is a well-known activator of SIRT1, which is thought to mediate the beneficial effects of caloric restriction [7, 8, 49], so it is possible that resveratrol may be activating SIRT1 in the honey bee and influencing food consumption. Lastly, it should be noted that the decrease in food consumption in honey bees is not likely related to factors of palatability since honey bees showed no adverse behavior to resveratrol in prior trials.

### Resveratrol, sirtuins, and the epigenome

The environmental conditions of early life, particularly nutrition, leave consequential signatures on the epigenome that can span across generations [50-52]. In the honey bee, differences in nutrition during development lead to the formation of distinct castes in genetically identical individuals. These differences in nutritional input in the honey bee can be traced to changes in DNA methylation [53]. Resveratrol is a nutritional supplement and an activator of SIRT1, a histone deacetylase [2]. SIRT1 can influence DNA methylation in mammalian cells [54]. In mammals, aging is associated with a loss of DNA methylation and declining transcriptional control of methyltransferases [55, 56]. This loss of DNA methylation during aging may contribute to the signature genomic instability and shortened telomeres that characterize aging cells [57, 58]. Recently, a study demonstrated that SIRT1 represses a large set of genes in the mouse genome and promotes repair of DNA strand breaks [59]. This particular study also determined that increased SIRT1 promotes survival in the mouse and reduces transcriptional abnormalities associated with aging. Thus, it is possible that resveratrol and its subsequent activation of SIRT1 may promote genomic stability and a delay in aging via similar mechanisms.

### Resveratrol, sirtuins, and the TOR network

In addition to activating sirtuins, resveratrol has been shown to inhibit several members of the Target of Rapamycin (TOR) network in mammals, e.g. AMP-activated protein kinase (AMPK), Phosphatidylinositol 3-kinase (PI3K), and Mitogen-activated protein kinase (MAPK) [17, 60-62]. Suppression of TOR activity is known to slow aging in yeast, worms, and flies [6, 63-65]. Notably, rapamycin also extends the lifespan of fruit flies and mice [66, 67]. A recent study of mammalian cells demonstrated that resveratrol preserved their proliferative capacity and inhibited S6 kinase phosphorylation, thereby eliciting an indirect repression of mTOR activity [68]. This result is similar to the effect of rapamycin on mTOR in cells [69]. A separate cellular study revealed that resveratrol blocks autophagy via the inhibition of S6 kinase under nutrient-limited conditions [70]. In contrast, cells grown in nutrient-rich media supplemented with resveratrol showed an increase in autophagy [70]. Furthermore, the negative regulation of S6 kinase homologs produces anti-aging effects in yeast [71-73] and fruit flies [64, 74]. It is possible that sirtuins and TOR may be part of the same caloric restriction pathway in mammals since both caloric restriction and TOR inhibition lead to increased expression of a key sirtuin regulator [75].

The molecular connections between resveratrol, sirtuins, the epigenome, and TOR remain unexplored in the honey bee. However, if some of the aforementioned links are conserved in this species, they may explain some of the organismal-level changes observed in this study.

### Conclusion

In summary, we demonstrated that resveratrol significantly affected gustatory responsiveness and prolonged lifespan in wild-type honey bees under normal oxygen conditions. However, the enhanced lifespan effect of resveratrol was abolished under hyperoxic conditions. Moreover, resveratrol had a satiety effect on honey bees and reduced their food consumption. These findings support the hypothesis that the lifespan extension effects of resveratrol are evolutionarily conserved.

### Future work

Our subsequent projects in honey bees will focus on using pharmacological agents to explore whether there is a SIRT1-dependence for the lifespan and neurophysiological effects noted here.

## MATERIALS AND METHODS

### Sample collection scheme and honey bees

Experiments were performed at Arizona State University in Tempe, AZ, USA. We utilized four genetically diverse wild type stock colonies (*Apis mellifera*) headed by queens of Californian commercial origin that were mated with multiple males. The colonies each had a single queen and several thousand workers. Each queen was caged onto a comb and allowed to lay eggs over a period of one day, with an additional day for proper acclimatization. This procedure made brood easy to track temporally and spatially.

To ensure robust experimental replication and manageable workloads, we employed a staggered honey bee collection scheme, which was repeated every week for a total of five weeks. This experimental design allowed for a predictable supply of age-matched newly emerged bees three times per week every Thursday, Friday, and Saturday beginning calendar week 44 and ending week 49. To collect newly emerged bees (0-24 h old), brood combs were placed in an incubator overnight at 34°C in a relative humidity of 65-70%. Upon emergence the following morning, bees were marked on the thorax with a designated paint color (Testors, Rockford, Illinois, USA) for identification and placed in a host colony. After four days, marked honey bees were recaptured and taken into the laboratory. We reasoned that newly emerged bees— which cannot feed themselves—should remain in the colony for the first days of life before transfer to a laboratory setting so that they could procure essential social provisions [76, 77].

### In-lab processing

We captured four-day-old honey bees at 9 AM every Monday, Tuesday, and Wednesday and then placed individuals into 7.0 × 3.5 × 3.5 cm plastic tubes. Then, we brought the honey bees to the laboratory (< 5 min transit time) where they were incubated at 4°C until movement was reduced. Next, we placed bees into wire mesh cages in groups of 30 and randomly assigned them to 0, 30, or 130 μM resveratrol treatment. Honey bees were fed and maintained in cages for five days until they reached the age of nine days. In the cages, honey bees had *ad libitum* access to water and a pollen-sucrose diet. Subsequently, we placed the cages containing treatment animals in an incubator that maintained optimal environmental conditions of 34°C and 65-75% relative humidity. Diets were freshly prepared each day for all treatment groups. After spending five days in cages, we tested each nine-day-old honey bee cohort (3 cohorts per week) for gustatory responsiveness and olfactory learning performance every Saturday, Sunday, and Monday for five weeks. All sensory and gustatory tests were performed on honey bees that had only experienced normoxia. After the completion of sensory and learning tests, we placed honey bees in either a hyperoxygenated (75% O2) or a normoxic (21% O2) environment, and then measured survivorship.

### Diet preparation

We prepared a liquefied diet of protein and carbohydrates consisting of 1.5 g of freshly ground pollen per 30 mL of 30% sucrose solution. Batches of this mixture were stored in frozen aliquots of 25 ml and thawed daily upon use. We solubilized resveratrol—which was kept in the dark at −20 °C— in molecular grade ethanol and added it to the pollen-sucrose diet (1:1000 dilution of resveratrol to liquid diet) daily. The daily addition of fresh resveratrol to the diet ensured optimal activity of the drug since it is known that resveratrol degrades in food medium after 24 h in 37° C [33]. Three concentrations of resveratrol (0, 30, 130 μM) were chosen as the basis for our treatment groups based on in vitro activation levels for *Drosophila*. The resveratrol we utilized is routinely tested for activity. We began administering resveratrol when honey bees were four-days-old because resveratrol works best when fed during early adulthood [4].

### Sensory and learning test preparation

Following a five-day resveratrol treatment in cages, approximately 30 bees per resveratrol group/day (3 days per week, 5 weeks total) aged nine days were prepared for sensory and learning performance tests. To ease handling and placement of bees into individual tubes, they were cooled in 4°C until reduced movement was detected. Honey bees were then restrained in small polyacryl holders using strips of duct tape. As with all laboratory tests, test bees were randomized so that experimenters were blind to treatment identity. Thereafter, bees were fed 2 μl of 30% sucrose solution and were placed in an incubator for a starvation period of two hours. The incubator maintained atmospheric oxygen at normoxic levels.

### Gustatory response measurements

To measure gustatory responsiveness, we utilized the proboscis extension response (PER). The criterion for a positive PER was complete extension of the proboscis. Nine-day-old honey bees under resveratrol or control treatment were stimulated over the antennae with water and six subsequent sucrose solutions in the following order: 0.1, 0.3, 1, 3, 10, 30%. We adhered to an inter-stimulus interval of two minutes to prevent sensitization and habituation. An overall index of performance, or gustatory response score (GRS), was calculated for all tested bees by using the sum of all PER to seven different stimuli (water and six sucrose solutions). A honey bee with a total score of 7 showed the highest level of sensory responsiveness, while a zero score indicated no responsiveness. Bees that failed to respond to the 30% sucrose stimulus were not included in the olfactory conditioning trials as this sucrose concentration was used as a reward in subsequent learning trials.

### Olfactory conditioning

In an effort to examine the whether resveratrol could enhance or improve brain function, we tested learning performance using a reward-based olfactory conditioning paradigm which we have previously published (Rascón & Amdam, in progress). Briefly, this method involves pairing an odor with a sucrose reward over six trials of conditioning to test associative learning performance. After each conditioning trial, we scored PER as binary variable via PER (i.e., response or no response). Once all conditioning trials took place, we tested the honey bees for odor generalization by presenting them with the unconditioned stimulus, cineole. This allowed us to test the bee's discrimination ability. Subsequently, we calculated a learning acquisition score based on conditioned responses. The score, with a numerical value between 0 and 5, was based on 5 conditioning trials and an additional trial that tested reaction spontaneity.

### Hyperoxic stress exposure

Oxygen exposure has been shown to impair honey bees faster and in a more controlled fashion than free-flight recapture setups. On their ninth day of life following gustatory and learning performance tests, honey bees were maintained in an incubator (HERAcell O_2_/CO_2_, Thermo Scientific) under an enriched oxygen atmosphere (75% O_2_), 34°C, and a relative humidity of 63±2%. Relative humidity was monitored by Hobo data loggers (Onset, MA, USA). Bees were individually housed in 1.5 mL Eppendorf tubes, each outfitted with a feeding port, breathing hole, and an opening for waste and defecation, as previously described [78]. Honey bees were fed 25 μL of the aforementioned protein-carbohydrate-resveratrol diet and were allowed to feed *ad libitum* through an easily accessible food-containing pipette tip. Feeding was verified to prevent starvation and/or caloric restriction, and thereby minimize survivorship effects not associated with treatment. Honey bees were continually fed their respective resveratrol diet mixtures until death.

### Survivorship measurements

The survivorship assay began when honey bees were nine-days-old and while maintained in either hyperoxia or normoxia. Survivorship was scored at four to five times per day until all subjects were dead. During these monitoring periods, bees were either observed dead or alive, and remaining live bees were transferred to fresh tubes in order to prevent bacterial and/or fungal growth. Individuals that appeared to have died due to accident (e.g. killed during routine transfers) were not included in the data analysis. Individual life spans were calculated using the frequency of bees alive at each temporal observation.

### Food consumption measurements

We measured individual food consumption in honey bees that were reared on ad libitum diets of protein-carbohydrate-resveratrol. Following an administration of 0 or 130 μM resveratrol treatment for five days, we prepared 50 nine-day-old honey bees per group for food consumption measurements, exactly as noted above for sensory and learning tests. After a starvation period of two hours, bees were allowed to feed until satiation. Diets were administered one microliter at a time and in this way we were able to quantify food consumption on an individual-level basis.

### Statistical analyses

Our gustatory responsiveness and learning performance data were non-normal as determined by normal probability plots, so we used non-parametric statistics for analysis. We used the Kruskal-Wallis ANOVA to assess overall treatment effects, and the Mann-Whitney U test as a *post hoc* test to examine differences between the groups. For analysis of survivorship data, we used Cox's F-test and the multi-sample survival test to verify longevity differences between treatment groups. The Kaplan-Meier estimator was employed to approximate the survival function for honey bee populations. Quantitative differences were considered statistically significant if alpha values were less than 0.05. All statistical analyses were carried out using *STATISTICA* 7.0 (Statsoft) and graphs were generated using R statistical software, version 2.12.1 (www.r-project.org).
